# RTEX: A novel framework for ranking, tagging, and explanatory diagnostic captioning of radiography exams

**DOI:** 10.1093/jamia/ocab046

**Published:** 2021-04-21

**Authors:** Vasiliki Kougia, John Pavlopoulos, Panagiotis Papapetrou, Max Gordon

**Affiliations:** 1 Department of Computer and Systems Sciences, Stockholm University, Stockholm, Sweden; 2 Division of Orthopaedics, Department of Clinical Sciences, Danderyd Hospital, Karolinska Institutet, Stockholm, Sweden

**Keywords:** deep learning, information storage and retrieval, diagnostic imaging, diagnostic captioning, computer-assisted diagnosis, explainability

## Abstract

**Objective:**

The study sought to assist practitioners in identifying and prioritizing radiography exams that are more likely to contain abnormalities, and provide them with a diagnosis in order to manage heavy workload more efficiently (eg, during a pandemic) or avoid mistakes due to tiredness.

**Materials and Methods:**

This article introduces RTEx, a novel framework for (1) ranking radiography exams based on their probability to be abnormal, (2) generating abnormality tags for abnormal exams, and (3) providing a diagnostic explanation in natural language for each abnormal exam. Our framework consists of deep learning and retrieval methods and is assessed on 2 publicly available datasets.

**Results:**

For ranking, RTEx outperforms its competitors in terms of *nDCG@k*. The tagging component outperforms 2 strong competitor methods in terms of F1. Moreover, the diagnostic captioning component, which exploits the predicted tags to constrain the captioning process, outperforms 4 captioning competitors with respect to clinical precision and recall.

**Discussion:**

RTEx prioritizes abnormal exams toward the improvement of the healthcare workflow by introducing a ranking method. Also, for each abnormal radiography exam RTEx generates a set of abnormality tags alongside a diagnostic text to explain the tags and guide the medical expert. Human evaluation of the produced text shows that employing the generated tags offers consistency to the clinical correctness and that the sentences of each text have high clinical accuracy.

**Conclusions:**

This is the first framework that successfully combines 3 tasks: ranking, tagging, and diagnostic captioning with focus on radiography exams that contain abnormalities.

## INTRODUCTION

**Figure 1.**
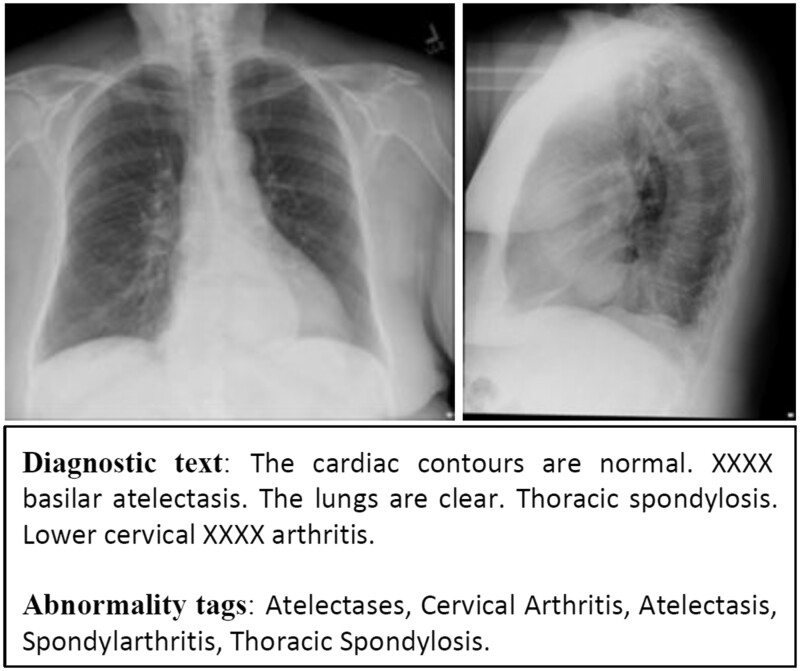
A posteroanterior/lateral chest radiography exam along with the corresponding human-authored DIAGNOSTIC TEXT from IU X-ray, and the abnormality tags. The “XXXX” is due to the de-identification process.

Medical imaging is the method of forming visual representations of the anatomy or a function of the human body using a variety of imaging modalities (eg, computed radiography, computed tomography, magnetic resonance imaging).^1^^,^^2^ In this article, we particularly focus on chest radiography exams, which contain medical images produced by x-rays. It is estimated that over 3 billion radiography exams are performed annually worldwide,^3^ making the daily need for processing and interpretation of the produced radiographs paramount. The daily routine of diagnostic radiologists includes the examination of radiographs for abnormalities or other findings, and an explanation of these findings in the form of a medical report per radiography exam.^4^ This is a rather challenging and time-consuming task, imposing a high burden both to radiologists and patients. For example, approximately 230 000 patients in England are waiting for over a month for their imaging test results,^5^ while 71% of the clinics in the United Kingdom report a lack of clinical radiologists.^6^ An example of a radiography exam is provided in Figure 1, consisting of 2 chest radiographs, the diagnostic text describing the medical observations on the radiographs, and a list of abnormality tags indicating the most critical observations in the exam.

While several methods have emerged that automatically detect abnormalities in radiographs^7^^,^^8^ or generate a diagnostic text,^9–12^ their solutions are hindered by 3 major challenges:

- **Screening and prioritization.** Radiologists have to examine a large amount of radiographs and write diagnostic reports, which is a demanding and time-consuming task. Current methods do not perform prioritization of the exams.
- **Clinically correct diagnostic captioning.** Existing diagnostic captioning models are not optimized in terms of clinical correctness, as they are trained on both normal and abnormal exams. This makes them less effective compared with being trained only on abnormal exams (see Results).
- **Explainability and clinical relevance.** On the one hand, system-generated visual explanations usually only function as means for highlighting image parts relevant to the diagnostic tags, without any textual explanation. On the other hand, diagnostic captioning methods can provide both a diagnosis and an explanation for the problem at hand; however, the produced reports are typically of low clinical correctness, as they are not optimized in terms of clinical relevance.^13^

We address these challenges by introducing a novel framework called RTEx. Our main contributions are summarized as follows:

- **Novelty.** RTEx provides 3 key functionalities: (1) ranking of abnormal radiography exams, prioritizing those likelier to include an abnormality from a large collection of normal and abnormal ones; (2) diagnostic tagging, generating a set of abnormality tags for the highly ranked radiography exams, trained on an independent set of abnormal ones; and (3) diagnostic captioning, the predicted tags are used by RTEx to provide a diagnostic text, serving as a clinically relevant explanation of the detected abnormal findings.
- Applicability and efficiency. We provide an empirical evaluation of RTEx, using 2 publicly available datasets of radiography exams.^14^^,^^15^ Our benchmarks assess the performance of RTEx on the ability to (1) rank abnormal radiography exams higher than normal ones, (2) produce the correct medical abnormality tags for abnormal exams, and (3) explain the reasoning behind the selection of the detected tags in the form of diagnostic text. Moreover, we perform a runtime experiment to demonstrate the time efficiency of RTEx.
- **Effectiveness and clinical accuracy.** Our experiments further demonstrate the effectiveness of RTEx against state-of-the-art competitors for the tasks of ranking, tagging, and captioning. In addition, human evaluation indicates that RTEx@X produces texts of high clinical accuracy, and when using predicted tags, it can produce text with higher clinical consistency.

### Related work

Automated screening of radiography exams is not a novel idea.^16–18^ Many works perform binary classification by employing pretrained convolutional neural networks (CNNs), eg, DenseNet-121^19^ and VGG-19,^20^ and report high scores.^21^^,^^22^ As they mention, these methods can also be used for exam prioritization. Especially when the number of exams is overwhelming, the employment of an automated method to exclude normal cases can lead to faster treatment of abnormal cases. Recently, pretrained CNNs were found to successfully distinguish normal cases from ones with pneumonia and COVID-19 (coronavirus disease 2019).^23^ The authors of the previously mentioned works noted that their models aim to ease the workload of radiologists, which is also an objective of our work. However, we propose a ranking of the exams based on their probability of being abnormal, rather than classifying exams as normal and abnormal, which has not been proposed by previous works. Also, a lot of research has been focused on labeling radiographs that are associated with a single abnormality,^24^^,^^25^ assuming that the problem is a priori known. This is not always the case, for example, when a new patient arrives for the first time to the clinic.

Another line of research, that of classifying multiple abnormality types, focuses on associating medical tags to radiographs. This task is addressed in the literature,^8^ as well as in the ImageCLEFmed Concept Detection competition that is held every year.^7^^,^^26–28^ In 2017, retrieval-based methods achieved the highest scores,^29^ while in 2018 onward, the best methods were deep learning classifiers.^30^ In 2019, first place was awarded to a DenseNet-121 CNN followed by a feedforward neural network (FFNN).^31^ The third-best method was a DenseNet-121 CNN encoder followed by a k-NN image retrieval approach. This work builds on top of the 2 best-performing methods (the second place was awarded to an ensemble of the 2 best-performing methods). The method ranked first in 2020 was an ensemble of the best-performing method of the previous year.^32^

Image captioning has been applied to medical images in order to assist clinicians in authoring diagnostic reports.^26^^,^^27^^,^^33–35^ The widely used architecture for this is encoder-decoder usually with visual attention,^9^^,^^36^ while incorporating predicted tags into the text generation process has been shown to achieve very good results.^37^ Image retrieval methods for diagnostic captioning can also achieve competitive performance,^11^^,^^38^ but using predicted tags in these approaches has not been examined before.

## MATERIALS AND METHODS

### The RTEx framework

**Figure 2.**
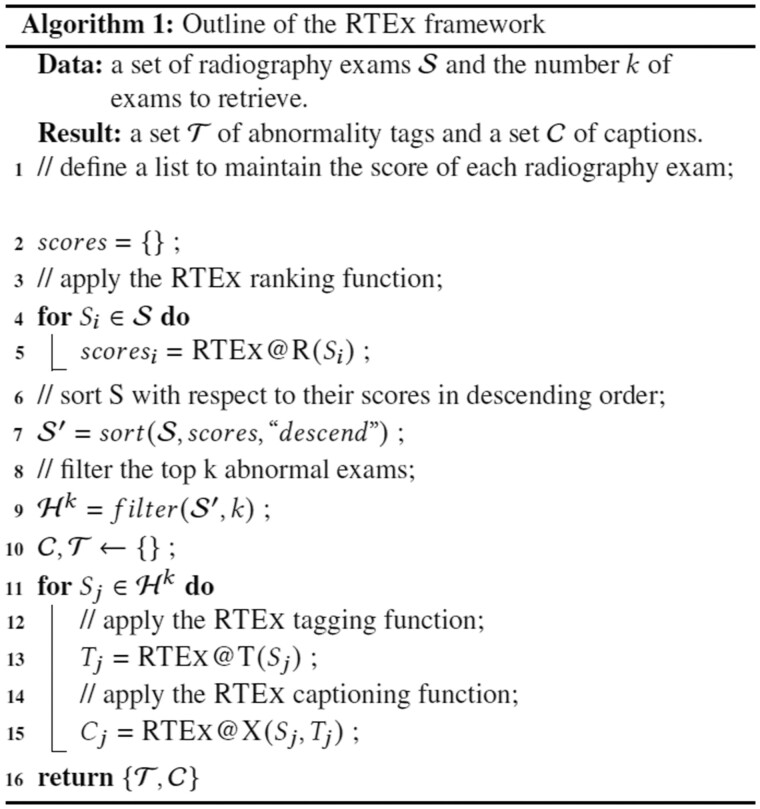
The algorithm of our RTEx framework.

**Figure 3.**
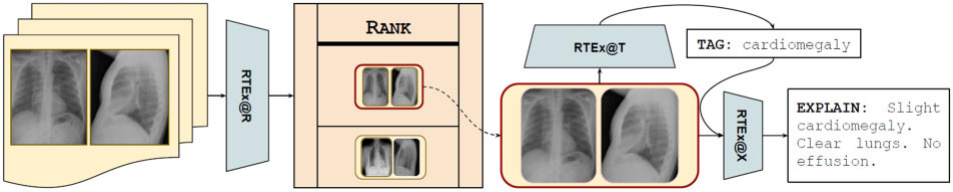
A depiction of our RTEx framework. It first ranks the radiography exams based on their probability (ie, using the radiographs of each exam) to include an abnormality. The highest ranked are tagged with abnormality terms and an explanatory diagnostic text is automatically provided to assist the expert.

We present the 3 stages of RTEx that are outlined in Figure 2, with an overview of the whole pipeline depicted in Figure 3.

#### RTEx@R: Ranking

**Figure 4.**
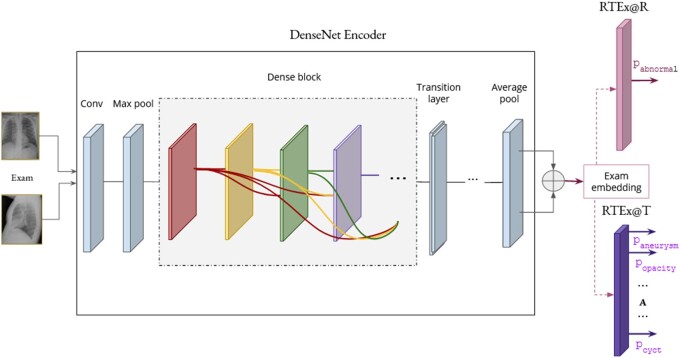
The architecture of RTEx@R and RTEx@T. They both take as input a radiography exam, employ DenseNet-121 to obtain an exam embedding, and feed it to a dense layer that serves as the classifier. RTEx@R outputs the probability of an exam to be abnormal. On the other hand, the input of RTEx@T is an abnormal radiography exam and the output consists of A binary nodes, where A is the total number of tags in the dataset. The nodes that yield probabilities higher than a defined threshold indicate the presence of the respective medical abnormalities.

For the first stage in our framework,. we implement an architecture, which we refer to as RTEx@R, shown in Figure 4. We employ the same visual encoder as in Rajpurkar et al.^39^ That is the DenseNet-121 CNN, which is followed by a FFNN. The input of the network comprises images of radiography exams, while the output is a score representing the probability that the exam in question is abnormal. First, both images of the exam are fed to DenseNet-121 (depicted inside the box in the center), and an embedding for each image is extracted from its last average pooling layer. These embeddings are concatenated to yield a single embedding for the radiography exam. Then, the exam embedding is passed to a FFNN with a sigmoid to return a score from 0 (normal) to 1 (abnormal).

#### RTEx@T: Diagnostic tagging

The second stage of RTEx comprises the assignment of a set of tags $T_{j}$ to a radiography exam $S_{j}\;\in \;H^{k}$, where $H^{k}$ is the set of the top *k* abnormal exams. Our method for addressing this task is called RTEx@T and is shown in Figure 4. It is similar to RTEx@R in that it uses the DenseNet-121 CNN encoder and an FFNN. But it differs in that the FFNN has 1 output and 1 sigmoid activation per abnormality tag in the dataset, leading to A different output nodes (the bottom right arrows in the figure). In effect, it returns a probability distribution over the abnormality tags and if the probability of an abnormality tag (ie, its respective node) exceeds a learned threshold, then the tag is assigned to the radiography exam.

#### RTEx@X: Diagnostic captioning

For the last stage of our framework (Figure 3), referred to as RTEx@X, we use the DenseNet-121 CNN encoder, calibrated for the task of diagnostic captioning. More specifically, each radiography exam in the database is encoded (offline) by our CNN to an embedding (ie, 2 image embeddings extracted from the last average pooling layer of the encoder, concatenated). Our CNN also encodes any new test exam. Then, the cosine similarities between the test embedding and all the training embeddings in the database are calculated and the most similar exam is retrieved from the database. Its diagnostic text is then assigned to the test exam. RTEx@X limits its search to training exams that have the exact same tags as the ones predicted (during the tagging stage) for the test exam. However, the whole database is searched when no exams exist with the same tags. We note that this method is the most efficient compared with its competitors (milliseconds instead of minutes).

### Datasets

#### IU X-ray

The IU X-ray^14^ is a collection of radiology exams that is publicly available through the OpenI (Open Access Biomedical Image Search Engine) (https://openi.nlm.nih.gov/). The dataset consists of 3995 radiology reports (1 report per patient) and 7470 frontal or lateral radiographs, with each report consisting of an “indication” (eg, symptoms), a “comparison” (eg, previous information about the patient), a “findings,” and an “impression” section. Each report contains 2 groups of tags. There are manual tags (a combination of MeSH codes [https://goo.gl/iDvwj2] and RadLex codes [http://www.radlex.org/]) assigned by 2 trained coders, each comprising a heading (disorder, anatomy, object, or sign) and subheadings (eg, “hiatal/large,” where “large” is an attribute). Also, each report is associated with tags, extracted automatically by Medical Text Indexer (MTI tags).^40^ An example exam is shown in Figure 1, in which it can be seen that the MTI tags are simple words or terms (eg, “Hiatus”).

For the ranking stage of our framework, each exam was labeled as abnormal, if 1 or more manual abnormality tags were assigned, and normal otherwise (the tag “normal” or “no indexing” was assigned). For the tagging stage of our framework, we employed the MTI codes because the manual codes do not explicitly describe the abnormality, but most often also include other information (eg, anatomical site). For the explanation stage, we employed the “findings” section. Also, in our experiments we used only exams with 2 images considering this to be the standard (1 frontal and 1 lateral radiograph) and excluded the rest. We also discarded exams that did not have a “findings” section. This resulted in 2790 exams, from which 1952 are used for training, 276 for validation, and 562 for testing (we used the same split as in Li et a).^9^^,^^33^ The class ratio in the dataset is slightly imbalanced, with 39% normal radiology exams. Abnormal exams are assigned with 3 tags on average, while the most frequent tag is “degenerative change.” The length of the diagnostic text in each report is 40 words on average. For the normal exams the diagnostic text can be exactly the same for many different patients (eg, 29 exams), while the most frequent abnormal text appeared exactly the same in 7 exams.

#### MIMIC-CXR

The MIMIC-CXR dataset comprises 377 110 chest radiographs associated with 227 835 exams that come from 64 588 patients of the Beth Israel Deaconess Medical Center (MIMIC-CXR v2.0.0 [https://mimic-cxr.mit.edu/]). As in IU X-ray, reports in MIMIC are organized in sections, while some reports include additional sections ,”as “history,” “examination,” or “technique,” but not in a consistent manner.^15^ The current version of the dataset does not contain the initial labels, so we reproduced them by applying the CheXpert disease mention labeler^41^ on the reports as described in Johnson et al.^15^ CheXpert classifies texts into 14 labels (13 diseases and “no finding”), each as “negative,” “positive,” or “uncertain” for a specific text. We treated those labeled uncertain as positive. For the ranking step, we labeled exams as normal when the “no finding” label was assigned. In total, there are 40 306 exams with 2 images that correspond to 29 482 patients. After removing 11 exams that did not have a “findings” section, which we used for the explanation stage of RTEx, we split the dataset to 70% (training), 10% (validation), and 20% (test) with respect to patients. For our experiments we randomly kept 1 exam per patient and sampled 2300 patients from the training set, 300 from the validation set, and 650 from the test set, with 68% of this final dataset consisting of normal exams. Each abnormal exam has 2 labels on average, while the most common label is “pneumonia.” The average diagnostic text length is 55 words. Many normal exams have the same diagnostic text, eg, the most common normal caption appears in 53 exams. Considering only the abnormal exams the most frequent caption appears 4 times.

### Experimental setup

#### Ranking and tagging

For the first 2 steps of RTEx we investigated one baseline, referred to as RANDOM. RTEx@R was also benchmarked against RTEx@T by using the maximum probability from the probability distribution over the tags as the abnormality probability. For the tagging stage, RTEx@T was compared with 2 competitors, referred to as CNN+NN and CNN+KNN. RTEx@R and its competitors are trained on both normal and abnormal exams, while at the tagging stage, the methods are trained only on abnormal exams. Next, we describe the baseline and the 2 tagging methods.

**RANDOM.** This is a baseline method used both for ranking and tagging and simulates the case in which no screening is performed. For the ranking task, it randomly returns a number serving as the abnormality probability. For tagging, it simply assigns a set of random tags from the training set. The number of tags assigned is the average number of tags per training exam.

**CNN+NN.** This method employs a DenseNet-121 CNN^19^ encoder, pretrained on ImageNet and fine-tuned on our datasets (IU X-ray or MIMIC-CXR). CNN+NN encodes all images (from the training and test sets) and concatenates the obtained representations for each radiograph in an exam, to yield a single representation per exam. Then, for each test representation, the cosine similarity against all the training representations is computed and the nearest exam is returned. The abnormality tags of the nearest exam are returned and assigned to the test exam.

**CNN+KNN.** This method is an extension of CNN+NN that uses the *k*-most similar training exams to compute the tags $T_{j}$ for exam $S_{j}$. To constrain the number of returned tags ($T_{j}$), only the r most frequent tags of the k exams are held. Moreover, we set r to be the average number of tags per exam of the particular k retrieved exams. We observe that CNN+KNN is considered a very strong baseline for tagging. It was ranked third in a recent medical tagging competition.^31^ The first 2 methods are RTEx@T (see RTEx@T: Diagnostic Tagging) and an ensemble of CNN+KNN and RTEx@T, respectively.

#### Diagnostic captioning

We benchmarked 3 competitors for the task of diagnostic captioning showing the benefits of RTEx@X in terms of clinical correctness.

**S&T** was introduced by Vinyals et al^42^ for image captioning and is only applicable for the stage of diagnostic captioning. As the encoder of the S&T architecture, we employ the DenseNet-121^19^ CNN, which is used to initialize a long short-term memory recurrent neural network decoder.^43^ A dense layer on top outputs a probability distribution over the words of the vocabulary, so that the decoder generates a word at a time. The word generation process continues until a special “end” token is produced or the maximum caption length is reached.

**S&T+** extends S&T (also applicable solely to diagnostic captioning) so that the generated text explains the predicted tags. Hence, after the encoding phase and prior to the decoding phase (before the generation of the first word), the tags are provided to the decoder, as if they were words of the diagnostic text; similar to *teacher* forcing.^44^ Because the decoder is an recurrent neural network, this acts as a prior during the decoding that will follow.

**ETD** follows a tag and image constrained Encoder-Decoder architecture. A DenseNet-121 CNN^19^ yields one visual embedding per exam. The decoder is a long short-term memory constrained from the visual embedding and the tags that were assigned to the exam during the tagging step.

For all the text generation methods mentioned previously, we preprocessed the text by tokenizing, lowercasing the words, and removing digits and words with length 1. We used the Adam optimizer^45^ everywhere with initial learning rate 10e-3. RTEX@T and RTEX@R were trained using binary cross-entropy loss and employed a learning rate reducing mechanism.^39^

## RESULTS

**Figure 5.**
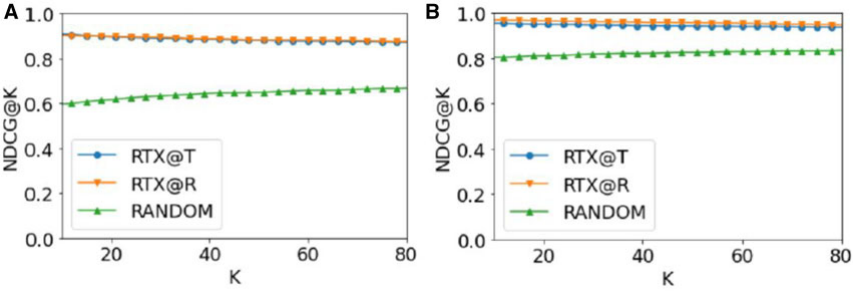
nDCG@K of all methods for the task of ranking radiography exams based on the probability of abnormality for (A) MIMIC-CXR and (B) IU X-ray. We used bootstrapping (1000 samples of 100 exams each) and report the average value. K varies from 10 to 80 and moving average was used with a window of 5. We observe that RTEX@R outperforms its competitors for both datasets.

The evaluation of the ranking methods was performed in terms of *nDCG@k*, with a varying *k*. We also used *Precision@k*, but preliminary experiments showed that this measure correlates highly with *nDCG@k.*Figure 5 depicts the performance of the methods. We used bootstrapping, sampling 100 exams at a time, varying *k* from 10 to 80 radiography exams. RANDOM is outperformed by both competitors, while RTEx@R is the overall winner for both datasets.

**Figure 6.**
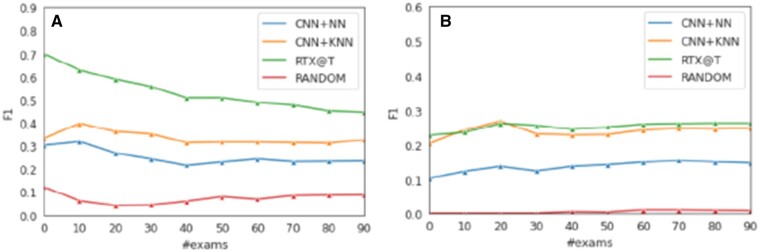
F1 of diagnostic tagging methods, on the top 100 ranked radiography exams. The cases were ranked by RTEx@R, based on their abnormality probability for for (A) MIMIC-CXR and (B) IU X-ray. We observe that RTEx@T is the winner for both datasets, with CNN+KNN being the second best by up to a factor of 2 for MIMIC-CXR.

Tagging methods were evaluated in terms of *F1@k*. During this step we assume that the radiography exams are already ranked based on an abnormality probability. Thus, we evaluate various methods with respect to their ability to correctly detect the abnormality tags. We used the top-*k* abnormal cases (ranked by RTEx@R) to compute the Macro F1 score (macro averaging across exams) between their predicted and their gold tags, which is also the standard measure of a recent competition on medical term tagging.^7^ As it can be seen in Figure 6, RTEx@T outperforms the 2 competitors in both datasets, with the second best being CNN+KNN, with a difference of up to a factor of 2 for MIMIC-CXR.

Evaluation of system-produced diagnostic texts was undertaken using word-overlap and clinical correctness measures. The most common word-overlap measures in diagnostic captioning are BLEU^46^ and ROUGE-L.^47^ BLEU is precision-based and measures word n-gram overlap between the produced and the ground truth texts. ROUGE-L measures the ratio of the length of the longest common n-gram shared by the produced text and the ground truth texts, to either the length of the ground truth text (ROUGE-L Recall) or the length of the generated text (ROUGE-L Precision). We employ the harmonic mean of the 2 (ROUGE-L F-measure). For the implementations of BLEU and ROUGE-L, we used respectively sacrebleu (https://github.com/mjpost/sacrebleu/blob/master/sacrebleu/sacrebleu.py) and MSCOCO (https://github.com/salaniz/pycocoevalcap/tree/master/rouge). To evaluate the clinical correctness, following the work of,^11^ we used the CheXPert labeler^41^ to extract labels from both the ground truth and the system-produced diagnostic texts. Clinical precision is then the average number of labels shared between the ground truth and system-generated texts, to the number of labels of the latter. Similarly, clinical recall is the average number of labels shared between the ground truth and system-generated texts, to the number of labels of the former. However, we note that these measures have limitations, since CheXpert shows only if an abnormality is mentioned (and not for example its location) and decides only for 14 labels.

Table 1 provides the results for the task of diagnostic captioning. We considered as ground truth the correct reports and as predicted captions the system-produced diagnostic texts. Our RTEx@X outperforms all methods in terms of clinical precision and recall. On the one hand, generative models achieve higher word-overlap scores, mainly because they learn to repeat common phrases that exist in the reports. On the other hand, retrieval methods assign texts that are written from radiologists, so they have a higher clinical value. When training S&T on all exams (S&T@ALL), using both normal and abnormal cases, clinical precision and recall decrease in both datasets. By contrast, the performance in terms of word-overlap measures (BLEU and ROUGE-L) was slightly improved overall, probably because the decoder is now better in generating text present in normal reports, which however is also present in abnormal reports (see Figure 1).

**Table 1.** The results of our explanatory captioning phase, evaluated with BLEU, ROU, CP, and CR

Dataset	Model	BLEU	ROU	CP	CR
MIMIC- CXR	S&T@ALL	7.8	25.7	0.080	0.118
	S&T	8.2	25.2	0.208	0.151
	S&T+	**9.8**	**26.2**	0.081	0.117
	ETD	6.9	25.5	0.171	0.144
	RTEX@X	5.9	20.5	**0.229**	**0.284**
IU X-ray	S&T@ALL	6.9	23.6	0.118	0.088
	S&T	6.5	23.0	0.153	0.113
	S&T+	9.5	23.4	0.085	0.071
	ETD	**10.0**	**26.7**	0.131	0.124
	RTEX@X	5.5	20.2	**0.193**	**0.222**

Clinical correctness decreases when S&T is trained also on normal exams (S&T@ALL). Our RTEX@X outperforms all other methods in CP and CR.

CP: clinical precision; CR: clinical recall; ROU: ROUGE-L.

Αs a final benchmark, we calculated the runtime of RTEx on ranking, tagging, and captioning on 500 randomly selected radiography exams from our IU X-ray test set. Ranking lasted 19.78 seconds. Producing tags and diagnostic texts for the top 100 ranked exams lasted 19.43 seconds. All 100 top-ranked exams in this experiment were abnormal. Note that an experienced radiologist needs 2 minutes on average^6^ for reporting a radiography exam, hence 200 minutes for 100 exams. The experiment was performed on a 32-core server with 256GB RAM and 4 GPUs.

**Repeatability.** For repeatability purposes, the code for the best performing pipeline of RTEx is available on github (https://github.com/ipavlopoulos/rtex.git).

**Qualitative analysis.** In order to assess the benefit of using the predicted tags in captioning, we gave 10 randomly selected exams to M.G. (M.G. is one of the authors and a medical practitioner assessing a large number of radiology exams per day.). M.G. was also provided with the 10 ground truth captions, 10 captions produced by RTEx@X, and 10 captions produced by RTEx@X without using any predicted tags. M.G., who did not have any prior information about which method produced each caption, assigned a score to each caption based on its clinical accuracy, from 1 (clinically inaccurate) to 5 (clinically accurate). Interestingly, by removing the tag constraint mechanism, the mean clinical accuracy remained the same, but its standard deviation was doubled, in effect leading to more inconsistent results. The outcome of this experiment suggests that the tags produced by RTEx@T have a detrimental effect on the clinical consistency of the produced captions.

We also performed human evaluation by asking 2 evaluators to examine the radiographs and identify factual errors in the RTEx@X-produced captions, eg, errors in the presence/absence or the characteristics of an abnormality (the evaluators are Dana Kokey and Anastasiia Iushina, who are medical experts) (Table 2). Each evaluator was provided with 20 exams, comprising the 10 top RTEx@R-ranked exams (set C) and 10 randomly chosen exams (distinct among the evaluators, sets S1 and S2). For the C set (49 sentences), we found an interannotator agreement (Cohen’s kappa) of 0.6 and a percentage agreement between the 2 evaluators of 85.7%. In Table 3, we report some of the results of the human evaluation. For more details, see the Supplementary Appendix.

**Table 2.** Results of the human evaluation for 5 captions produced by RTEx@X, randomly selected from the evaluation set

Caption Produced by RTEx@X	Sentence(s) With Factual Errors
**[S1]** The heart pulmonary xxxx and mediastinum are within normal limits. **[S2]** There is no pleural effusion or pneumothorax. **[S3]** There is no focal air space opacity to suggest a pneumonia. **[S4]** There are minimal degenerative changes of the spine.	**None**
**[S1]** There is hyperinflation. **[S2]** There is some subtle scarring in the lateral right base. **[S3]** There is no pleural effusion or pneumothorax. **[S4]** The heart is not significantly enlarged. **[S5]** There are atherosclerotic changes of the aorta. **[S6]** Arthritic changes of the skeletal structures are noted.	**[S1]**
**[S1]** The cardiomediastinal contours are within normal limits. **[S2]** Pulmonary vasculature is unremarkable. **[S3]** There is no focal airspace opacity. **[S4]** No pleural effusion or pneumothorax is seen. **[S5]** There is a stable calcified granuloma in the infrahilar right lung. **[S6]** There are mild degenerative changes along the thoracic spine. **[S7]** No acute bony abnormality is identified.	**[S5]**
**[S1]** Lungs are hyperexpanded. **[S2]** No infiltrates or masses in the lungs. **[S3]** Heart size normal.	**[S2]**
**[S1]** Stable normal cardiomediastinal silhouette. [**S2]** Bilateral calcified hilar perihilar lymph xxxx. **[S3]** Left lateral lung calcified granuloma. **[S4]** Lungs are grossly clear without focal consolidation pleural effusion or pneumothorax. **[S5]** Stable degenerative changes of the thoracic spine. **[S6]** No acute osseous abnormality.	**[S2, S3]**

For each caption we report the sentence(s) that contain at least 1 factual error.

**Table 3.** Micro- and macro-averaged clinical accuracies for the set of the top 10 ranked exams (C) that both experts evaluated, and the 2 sets of randomly selected exams (S1 and S2) evaluated by the first expert (E1) and the second expert (E2), respectively

Set	Schema	Clinical Accuracy	
		Micro	Macro
C	Per rater	E1: 0.755	E1: 0.714
		E2: 0.776	E2: 0.722
	Strict voting	0.837	0.788
S1	—	0.803	0.791
S2	—	0.816	0.799

For C, we calculated the accuracy per rater, as well as the accuracy considering a sentence false when both raters identified it as false (strict voting).

## DISCUSSION

In Table 4, we present the strengths and limitations of RTEx based on evaluation results and discussion with experts. Human evaluation of the RTEx@X-produced captions showed that the tag constraint mechanism offers consistency to their clinical accuracy. Also, 2 experts assessed the errors in produced sentences. Based on the results, RTEx@X achieved a micro clinical accuracy of 0.837, which is a considerably high score given the difficulty of the task. Higher clinical accuracy of randomly selected against top-ranked exams showed that the latter are harder due to their complexity and highlights the importance of the prioritization step.

**Table 4.** The strengths and limitations of RTEx as they infer from manual assessments, showing the advantages of our system when applied on clinical setting

Strengths	Limitations
The tagging and captioning components are trained on the prioritized abnormal exams, achieving **high clinical correctness**. Employing the tags for captioning leads to better **clinical consistency** of produced captions based on human evaluation. The inter-annotator agreement between 2 medical experts assessing the errors in produced sentences is 0.6. This low score implies that this is a **difficult task**, and a system should not be expected to score more than the percentage agreement between 2 experts, which we calculated (micro averaged) to be 85.7%. **High clinical accuracy**. Sentence-level human evaluation shows that 83.7% of the sentences produced by RTEx@X are correct, while the estimated uppermost threshold is 85.7%. This means that 83.7% of the sentences produced by RTEx@X would be in accordance with what an expert would say. In a clinical setting, these sentences could provide important evidence to an expert. Top-ranked exams achieve less clinical accuracy than the rest. This indicates that exams prioritized as more likely to contain an abnormality are harder to examine and make a diagnosis. RTEx@R prioritization step can be used to send the top-ranked exams, along with explanations (provided by RTEx@T & RTEx@X), to physicians with higher expertise. This could verify that hard exams would not reach the eyes of inexperienced or tired physicians, hence **reducing the probability of a medical error**.	Ranking prioritizes exams that are most likely to be abnormal but disregards the urgency/severity of an abnormality or the history of a patient. However, the clinical patient history and the severity of abnormalities do not exist in the current publicly available medical datasets, even though they are very important in a clinical setting in order to prioritize patients that need immediate care. Although RTEx is in principle applicable to any condition or type of exams, we only considered chest radiographs in this work, mainly due to their public availability. Our analysis is not based on an extremely large dataset, such as the one that could exist in a hospital in real world. However, we note that in large databases more duplicates are likelier to exist (patients with very similar conditions), and thus our retrieval-based RTEx@X component would perform even better.

We note that there are currently some limitations regarding the clinical applicability of RTEx (see Table 4). First, ranking is not based on the urgency/severity of an abnormality because this information is not available in the existing publicly available datasets. Also, RTEx has not been tested on larger datasets that are closer to a real-world scenario and other imaging exams. However, larger datasets could benefit our retrieval component, which we plan to extend by experimenting with retrieval-augmented generation ^48^ that achieves state-of-the-art results in language generation tasks. We argue that the strengths of our system show its clinical utility and importance despite the discussed limitations, which we will address with further experiments, directly passing the RTEx@R output to RTEx@T/X and longitudinal expert evaluation, with RTEx deployed in a hospital.

## CONCLUSIONS

We introduced a novel framework that can be used for (1) ranking radiography exams based on the probability of containing an abnormality, (2) producing diagnostic tags using abnormal exams for training, and (3) providing diagnostic text produced based on both radiographs and tags, as means of explaining the predicted tags. This is an important step for practitioners to prioritize cases with abnormalities. Our framework can be further used to predict abnormality tags and complement them with an automatically suggested explanatory diagnostic text to guide the medical expert. We experimented with 2 publicly available datasets showing that our ranking and tagging components outperform 2 strong competitors and a baseline. Our diagnostic captioning component achieves high clinical precision and recall, and human evaluation demonstrates the benefit of employing tags for producing text of higher clinical consistency. We also demonstrated that limiting training data to only abnormal exams improves the clinical correctness of the produced text. Additional sentence-level human evaluation showed that the sentences have high clinical accuracy. Future work includes deployment to a hospital, so that we can experiment with larger datasets and other imaging modalities.

## FUNDING

This work was partly supported by the VR-2016-03372 Swedish Research Council Starting Grant and by the EXTREMUM project of the Digital Futures framework.

## AUTHOR COTRIBUTIONS

The formulation of the problem was done by JP. The methods of RTEx and their competitors were implemented by VK. The experimental evaluation was performed by VK. JP, PP, and VK participated equally in the writing of the manuscript. MG provided consulting and his medical expertise. All authors provided feedback and read and approved the final version of the manuscript.

## SUPPLEMENTARY MATERIAL

Supplementary material is available at *Journal of the American Medical Informatics Association* online.

## Supplementary Material

ocab046_Supplementary_Data

## References

[1] Suetens P. *Fundamentals of Medical Imaging* . London, United Kingdom: Cambridge University Press; 2009.

[2] Aerts HJ , Velazquez ER, Leijenaar RT, et al Decoding tumour phenotype by noninvasive imaging using a quantitative radiomics approach. Nat Commun 2014; 5: 4006. 24892406 10.1038/ncomms5006 PMC4059926

[3] Krupinski EA. Current perspectives in medical image perception. Atten Percept Psychophys 2010; 72 (5): 1205–17. 20601701 10.3758/APP.72.5.1205 PMC3881280

[4] Monshi MMA , Poon J, Chung V. Deep learning in generating radiology reports: a survey. Artif Intell Med 2020; 106(: 101878. 32425358 10.1016/j.artmed.2020.101878 PMC7227610

[5] Royal College of Radiologists. Clinical radiology UK workforce census 2015 report, 2016. https://www.rcr.ac.uk/publication/clinical-radiology-uk-workforce-census-2015-report. Accessed September 1, 2020.

[6] Royal College of Radiologists. Clinical radiology UK workforce census 2019 report, 2019. https://www.rcr.ac.uk/publication/clinical-radiology-uk-workforce-census-2019-report. Accessed September 1, 2020.

[7] Pelka O , Friedrich CM, de Herrera AGS, et al Overview of the ImageCLEFmed 2019 concept detection task. CLEF2019 Working Notes 2019; 2380.

[8] Baltruschat IM , Nickisch H, Grass M, et al Comparison of deep learning approaches for multi-label chest X-ray classification. Sci Rep 2019; 9 (1): 1–10. 31011155 10.1038/s41598-019-42294-8 PMC6476887

[9] Jing B , Xie P, Xing E. On the automatic generation of medical imaging reports. In: *Proceedings of the 56th Annual Meeting of the Association for Computational Linguistics (Volume 1: Long Papers)*; 2018: 2577–86.

[10] Li Y , Liang X, Hu Z, et al Knowledge-driven encode, retrieve, paraphrase for medical image report generation. *Proceedings of the Association for the Advancement of Artificial Intelligence*; 2019: 6666–73.

[11] Liu G , Hsu T-MH, McDermott M, et al Clinically accurate chest x-ray report generation. arXiv, doi: https://arxiv.org/abs/1904.02633, 29 Jul 2019, preprint: not peer reviewed.

[12] Kougia V , Pavlopoulos J, Androutsopoulos I. A survey on biomedical image captioning. In: *Proceedings of the Second Workshop on Shortcomings in Vision and Language*; 2019: 26–36.

[13] Bluemke D , Moy L, Bredella MA, et al Assessing radiology research on artificial intelligence: A brief guide for authors, reviewers, and readers–from the *Radiology* editorial board. *Radiology* 2020; 294 (3): 487–9. 10.1148/radiol.2019192515 31891322

[14] Demner-Fushman D , Kohli MD, Rosenman MB, et al Preparing a collection of radiology examinations for distribution and retrieval. J Am Med Inform Assoc 2016; 23 (2): 304–10. 26133894 10.1093/jamia/ocv080 PMC5009925

[15] Johnson AEW , Pollard TJ, Berkowitz S, et al MIMIC-CXR: A large publicly available database of labeled chest radiographs. arXiv, doi: https://arxiv.org/abs/1901.07042, 14 Nov 2019, preprint: not peer reviewed. 10.1038/s41597-019-0322-0 PMC6908718 31831740

[16] Taguchi A. Triage screening for osteoporosis in dental clinics using panoramic radiographs. Oral Dis 2010; 16 (4): 316–27. 19671082 10.1111/j.1601-0825.2009.01615.x

[17] Jaeger S , Karargyris A, Candemir S, et al Automatic screening for tuberculosis in chest radiographs: a survey. Quant Imaging Med Surg 2013; 3 (2): 89–99. 23630656 10.3978/j.issn.2223-4292.2013.04.03 PMC3636475

[18] Oliveira LLG , Silva S A e, Ribeiro LHV, et al Computer-aided diagnosis in chest radiography for detection of childhood pneumonia. Int J Med Inform 2008; 77 (8): 555–64. 18068427 10.1016/j.ijmedinf.2007.10.010

[19] Huang G , Liu Z, Van Der Maaten L, et al Densely connected convolutional networks. In *Proceedings of the 2017 IEEE Conference on Computer Vision and Pattern Recognition*; 2017: 4700–8.

[20] Simonyan K , Zisserman A. Very deep convolutional networks for large-scale image recognition. arXiv, doi: https://arxiv.org/abs/1409.1556, 10 Apr 2015, preprint: not peer reviewed.

[21] Tang Y-X , Tang Y-B, Peng Y, et al Automated abnormality classification of chest radiographs using deep convolutional neural networks. NPJ Digit Med 2020; 3 (1): 70. 32435698 10.1038/s41746-020-0273-z PMC7224391

[22] Dunnmon JA , Yi D, Langlotz CP, et al Assessment of convolutional neural networks for automated classification of chest radiographs. Radiology 2019; 290 (2): 537–44. 30422093 10.1148/radiol.2018181422 PMC6358056

[23] Karim MR , Döhmen T, Rebholz-Schuhmann D, et al DeepCOVIDExplainer: Explainable COVID-19 diagnosis based on chest X-ray images. arXiv, doi: https://arxiv.org/abs/2004.04582, 6 Jun 2015, preprint: not peer reviewed.

[24] Roth HR , Lu L, Liu J, et al Improving computer-aided detection using convolutional neural networks and random view aggregation. IEEE Trans Med Imaging 2016; 35 (5): 1170–81. 26441412 10.1109/TMI.2015.2482920 PMC7340334

[25] Kong B , Zhan Y, Shin M, et al Recognizing end-diastole and end-systole frames via deep temporal regression network. In: Ourselin S, Joskowicz L, Sabuncu MR, Unal G, Wells W, eds. Medical Image Computing and Computer-Assisted Intervention - MICCAI 2016. Cham: Springer International Publishing; 2016: 264–72.

[26] Eickhoff C , Schwall I, de Herrera AGS, et al Overview of ImageCLEFcaption 2017 - the image caption prediction and concept extraction tasks to understand biomedical images. In: *CEUR Workshop Proceedings, CLEF2017 Working Notes*; 2017.

[27] de Herrera AGS , Eickhoff C, Andrearczyk V, et al Overview of the ImageCLEF 2018 caption prediction tasks. In: *CEUR Workshop Proceedings, CLEF2018 Working Notes*; 2018.

[28] Pelka O , Friedrich CM, de Herrera AGS, et al Overview of the ImageCLEFmed 2020 concept prediction task: medical image understanding. In: *CEUR Workshop Proceedings, CLEF2020 Working Notes*; 2020.

[29] Valavanis L , Stathopoulos S. IPL at ImageCLEF 2017 concept detection task. In: *CEUR Workshop Proceedings, CLEF2017 Working Notes*; 2017.

[30] Pinho E , Costa C. Feature learning with adversarial networks for concept detection in medical images: UA.PT Bioinformatics at ImageCLEF 2018. In: *CEUR Workshop Proceedings, CLEF2018 Working Notes*; 2018.

[31] Kougia V , Pavlopoulos J, Androutsopoulos I, AUEB NLP group at ImageCLEFmed caption 2019. In: *CEUR Workshop Proceedings, CLEF2019 Working Notes*; 2019.

[32] Karatzas B , Pavlopoulos J, Kougia V, et al AUEB NLP Group at ImageCLEFmed Caption 2020. In: *CEUR Workshop Proceedings, CLEF2020 Working Notes*; 2020.

[33] Hasan SA , Ling Y, Farri O, et al Overview of the ImageCLEF 2018 Medical Domain Visual Question Answering Task. In: *CEUR Workshop Proceedings, CLEF2018 Working Notes*; 2018.

[34] Abacha AB , Hasan SA, Datla VV, et al VQA-Med: Overview of the Medical Visual Question Answering Task at ImageCLEF 2019. In: *CEUR Workshop Proceedings, CLEF2019 Working Notes*; 2019.

[35] Abacha AB , Datla VV, Hasan SA, et al Overview of the VQA-Med Task at ImageCLEF 2020: Visual Question Answering and Generation in the Medical Domain. In: *CEUR Workshop Proceedings, CLEF2020 Working Notes*; 2020.

[36] Li Y , Liang X, Hu Z, et al Hybrid retrieval-generation reinforced agent for medical image report generation. In: *Proceedings of the 32nd International Conference on Neural Information Processing Systems*; 2018: 1537–47.

[37] Yuan J , Liao H, Luo R, et al Automatic radiology report generation based on multi-view image fusion and medical concept enrichment. In: *Proceedings of the International Conference on Medical Image Computing and Computer-Assisted Intervention;* 2019: 721–9.

[38] Boag W , Hsu T-M H, McDermott M, et al Baselines for chest x-ray report generation. In: *Proceedings of the Machine Learning for Health NeurIPS Workshop*; 2019: 126–40.

[39] Rajpurkar P , Irvin J, Zhu K, et al CheXnet: Radiologist-level pneumonia detection on chest x-rays with deep learning. arXiv, doi: https://arxiv.org/abs/1711.05225, 25 Dec 2017, preprint: not peer reviewed.

[40] Mork JG , Jimeno-Yepes A, Aronson AR. The NLM medical text indexer system for indexing biomedical literature. In: *Proceedings of the BioASQ Workshop*; 2013.

[41] Irvin J , Rajpurkar P, Ko M, et al CheXpert: A large chest radiograph dataset with uncertainty labels and expert comparison. In: *Proceedings of the Twenty-Third AAAI Conference on Artificial Intelligence*; 2019: 590–7.

[42] Vinyals O , Toshev A, Bengio S, et al Show and tell: A neural image caption generator. In: *Proceedings of the 2015 IEEE Conference on Computer Vision and Pattern Recognition*; 2015: 3156–64.

[43] Hochreiter S , Schmidhuber J. Long short-term memory. Neural Comput 1997; 9 (8): 1735–80. 9377276 10.1162/neco.1997.9.8.1735

[44] Goodfellow I, Bengio Y , Courville A. *Deep Learning* . Cambridge, MA: MIT Press; 2016.

[45] Kingma DP , Ba J. Adam: A Method for Stochastic Optimization. arXiv, doi: https://arxiv.org/abs/1412.6980, 30 Jan 2017, preprint: not peer reviewed.

[46] Papineni K , Roukos S, Ward T, et al BLEU: a method for automatic evaluation of machine translation. In: *Proceedings of the 40th Annual Meeting of the Association for Computational Linguistics ACL*; 2002: 311–8.

[47] Lin C-Y. ROUGE: A package for automatic evaluation of summaries. In: *Proceedings of the Workshop on Text Summarization Branches Out*; 2004: 74–81.

[48] Lewis P , Perez E, Piktus A, et al Retrieval-augmented generation for knowledge-intensive NLP tasks. arXiv, doi: https://arxiv.org/abs/2005.11401, 7 Dec 2020, preprint: not peer reviewed.

