# Supplementary material for: RTEX: A novel framework for ranking, tagging, and explanatory diagnostic captioning of radiography exams
Source: J Am Med Inform Assoc. 2021 Apr 21;28(8):1651–9. doi: 10.1093/jamia/ocab046 (PMC8324241; doi:10.1093/jamia/ocab046)
Supplement: ocab046_Supplementary_Data [file ocab046_supplementary_data.docx]

**Supplementary material**

**Inter-annotator agreement** We performed human evaluation on a set of 30 exams in total (159 sentences) by asking two evaluators to examine the radiographs and identify factual errors in the captions produced by RTEx@X, which concern errors in the presence/absence or the characteristics of an abnormality. This set consisted of the top-10 abnormal exams (as these were ranked by RTEx@R) and 20 exams that were randomly selected from the test set. Each evaluator was provided with 20 exams, comprising the 10 top ranked exams (we call this set C) and a set of 10 randomly chosen exams (distinct among the evaluators, which are the S1 and S2 sets). For the C set, which has 49 sentences in total, we found an inter-annotator agreement (Cohen’s Kappa) equal to 0.6. What this low agreement score implies is that this is a difficult task even for human experts and that a system should not be expected to score more than what an expert would achieve against another expert. We calculated this percentage agreement (micro averaged) to be 85.7%.

**Accuracy** We then calculated the clinical accuracy of the captions, based on the number of sentences evaluated as false by the two experts, i.e., sentences that contain at least one factual error. Both micro and macro clinical accuracy are reported. First, for the 10 common exams we calculated the accuracy per evaluator (E1 and E2), the accuracy assuming a sentence false when both evaluators annotated it as false (strict voting) and when at least one evaluator annotated it as false (lenient voting). The results are shown in Table 1. For the strict voting, which we trust more, RTEx@X achieves a micro clinical accuracy of 0.837. Given the difficulty of the task, where 85.7% is the estimated uppermost threshold, this is a considerably high score. This means that 83.7% of the sentences produced by RTEx@X would be in accordance with what an expert would say for the respective exams (if these sentences were produced by a fellow expert, that percentage would rise to 85.7%). In a clinical setting, these sentences could provide evidence to an expert, evidence that could have been missed otherwise. In that case any two experts who employ the tool are expected to reach higher inter-annotator agreement, compared to the 0.6 score that we computed in this study. We plan to undertake this experiment, along with a real-world deployment, in our future work.

| **Schema** | **Averaging** | **Clinical Accuracy** |
| --- | --- | --- |
| Per rater | Micro | **E1:** 0.755, **E2:** 0.776 |
|  | Macro | **E1:** 0.714, **E2:** 0.722 |
| Strict voting | Micro | 0.837 |
|  | Macro | 0.788 |
| Lenient voting | Micro | 0.694 |
|  | Macro | 0.648 |

**Table 1:** Clinical accuracy scores computed on C.

Table 2 presents the clinical accuracy score for each set of randomly selected captions, namely S1 and S2, evaluated by E1 and E2 respectively. We observe that the clinical accuracy is higher for S1 and S2, compared to the per rater accuracy for C. This is an indication that prioritized exams are harder, probably because their captions describe abnormalities and hence, have high textual diversity. Therefore, it is more difficult to produce a caption that is a perfect match to the gold one for the prioritized exams, than for the not prioritized, which contain more normal sentences that follow common templates. RTEx@R prioritization step can be used to send the top-ranked exams, along with explanations (provided by RTEx@T & RTEx@X), to physicians with higher expertise. This could verify that hard exams would not reach the eyes of inexperienced or tired physicians, hence reducing the probability of a medical error.

| **Set** | **Averaging** | **Clinical Accuracy** |
| --- | --- | --- |
| S1 | Micro | 0.803 |
|  | Macro | 0.791 |
| S2 | Micro | 0.816 |
|  | Macro | 0.799 |
| All | Micro | 0.755 |
|  | Macro | 0.795 |

**Table 2**: Clinical accuracy scores for S1, S2 and overall.

**Error analysis** A textual analysis of the errors detected by the experts showed that the most frequent words in the erroneous sentences are "calcified" according to E1 and "signs" according to E2. Both evaluators have the locality word "right" as the second most frequent one. This could mean that the system treats similarly right and left abnormalities (as left), but we note that despite this is treated as a factual error, that sentence could still be informative in principle.
